# Supplementary material for: Enterococcus faecalis autolysin EpaU binds the enterococcal polysaccharide antigen via its teichoic acid-like repeats and modulates c-di-AMP signaling
Source: mBio. 2026 Jun 15;17(7):e00988-26. doi: 10.1128/mbio.00988-26 (PMC13343894; doi:10.1128/mbio.00988-26)
Supplement: Supplemental Material — Figures S1 to S11; Tables S1 to S4. [file mbio.00988-26-s0001.pdf]

Supplemental Material

Enterococcus faecalis autolysin, EpaU, binds the enterococcal polysaccharide antigen via its teichoic acid-like repeats and modulates c-di-AMP signaling

Catherine T. Chaton[1], Nicholas R. Murner[2], Svetlana Zamakhaeva[2], Jeffrey S. Rush[1], Cameron W. Kenner[2*], Alexander E. Yarawsky[3], Lei Huang[4], Parastoo Azadi[4], Andrew B. Herr[5], Natalia Korotkova[1, 2#] and Konstantin V. Korotkov[1#]

[1]Department of Molecular and Cellular Biochemistry, University of Kentucky, Lexington, Kentucky, USA

[2]Department of Microbiology, Immunology and Molecular Genetics, University of Kentucky, Lexington, Kentucky, USA

[3]BioAnalysis LLC, Philadelphia, Pennsylvania, USA

[4]Complex Carbohydrate Research Center, University of Georgia, Athens, Georgia, USA

[5]Division of Immunobiology, Cincinnati Children's Hospital Medical Center, Cincinnati, Ohio; Division of Infectious Diseases, Cincinnati Children's Hospital Medical Center, Cincinnati, Ohio; Department of Pediatrics, University of Cincinnati College of Medicine, Cincinnati, Ohio, USA

#Address correspondence to Konstantin V. Korotkov, kkorotkov@uky.edu, or Natalia Korotkova, nkorotkova@uky.edu.

*Present address: Environmental Health & Safety, University of South Florida, Tampa, Florida, USA

Catherine T. Chaton and Nicholas R. Murner contributed equally to this work. Author order was determined alphabetically.

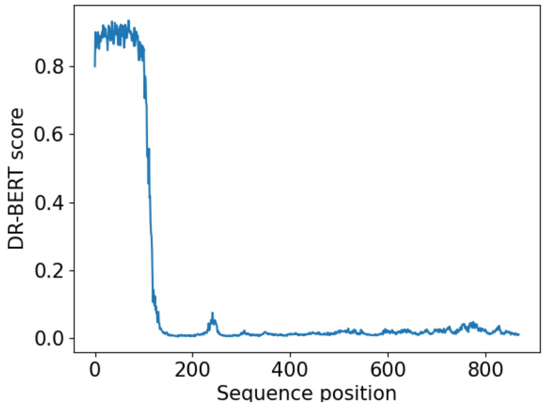

Figure S1. **The protein disorder prediction of EpaU^{V583} using DR-BERT.**
The protein disorder was predicted using a protein language model DR-BERT (1). Values above 0.75 are indicative of protein disorder. The signal peptide sequence is omitted.

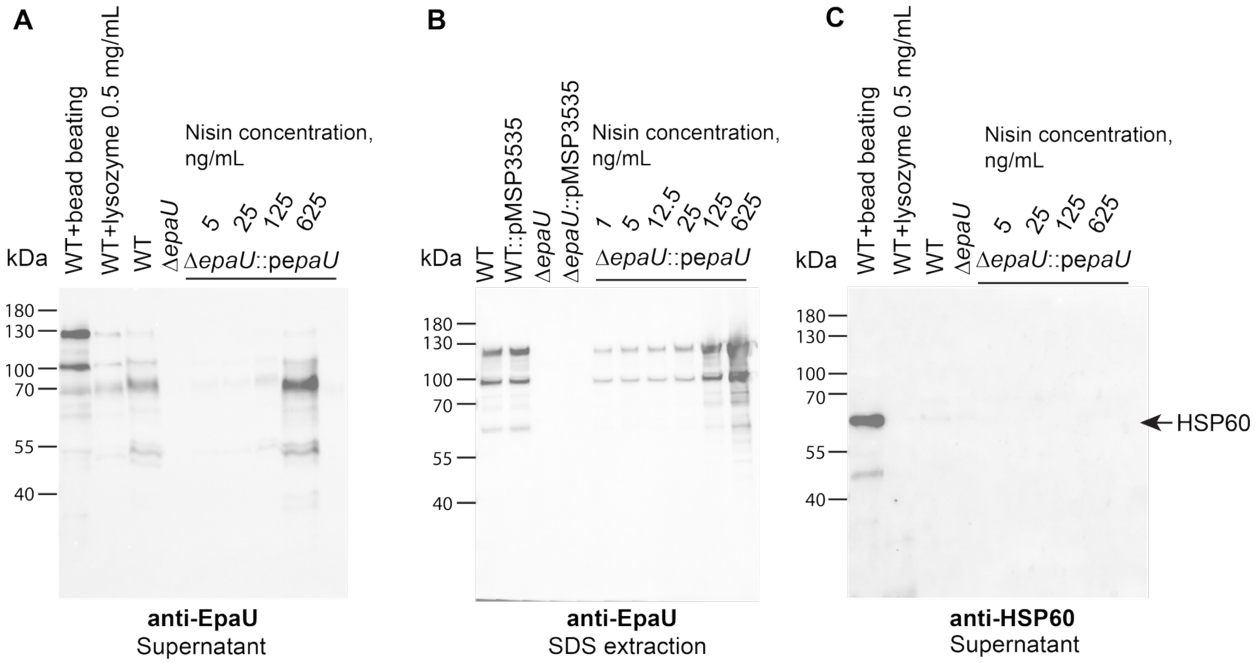

Figure S2. **Analysis of EpaU expression and the presence of HSP60 in the cell-free supernatant.**

(**A, B**) Immunoblots of EpaU precipitated from culture supernatants (A) and extracted from the cell surface with 2% SDS (B). (**C**) Proteins precipitated from culture supernatants were probed for heat-shock protein 60 (HSP60) using anti-HSP60 antibodies. EpaU expression in Δ*epaU*::p*epaU* was induced with varying concentrations of nisin. WT cells lysed by bead-beating or treated with lysozyme (0.5 mg mL^{-1}) were used as positive controls for HSP60 analysis. The experiments were performed independently two times and yielded the same results. A representative image from one experiment is shown.

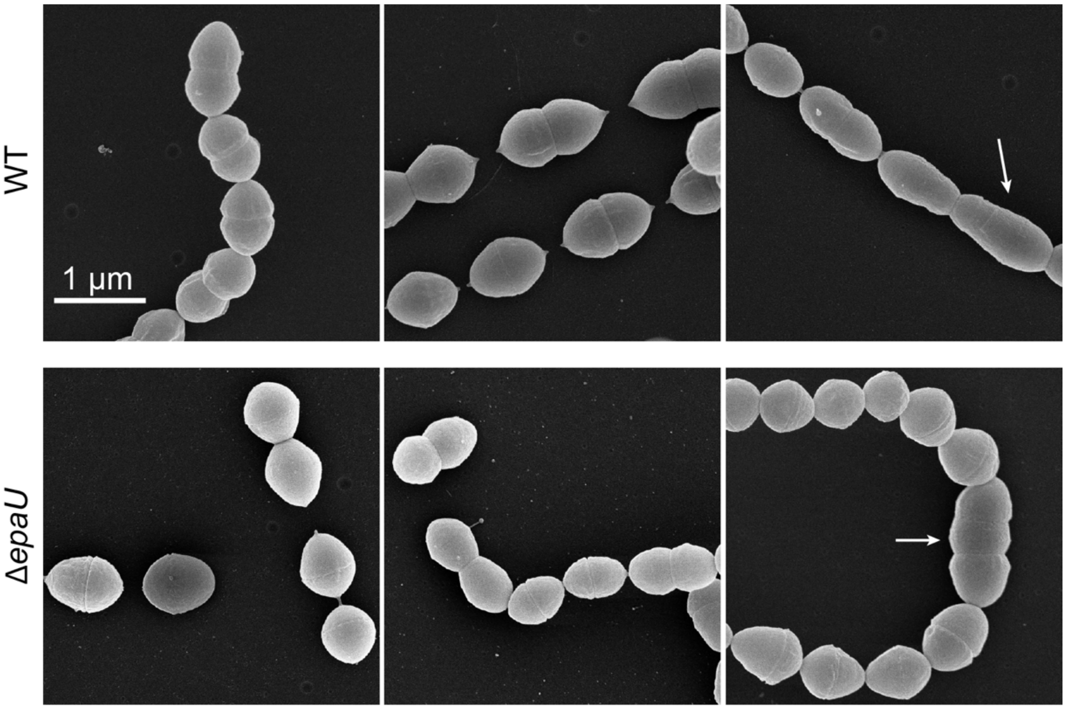

Figure S3. **Scanning electron micrographs of *E. faecalis* strains.**
Exponential phase bacterial cells were fixed, dehydrated, coated with platinum, and visualized by scanning electron microscopy. The majority of the cells in both strains are oval-shaped and connected into chains. White arrows denote cells with irregular cell division present in the WT and Δ*epaU* cells with similar frequency: 4.73% for WT (n=148) and 5.06% for Δ*epaU* (n=158), where n = number of cells observed. The imaging was performed independently three times and yielded the same results. Scale bar – 1 µm.

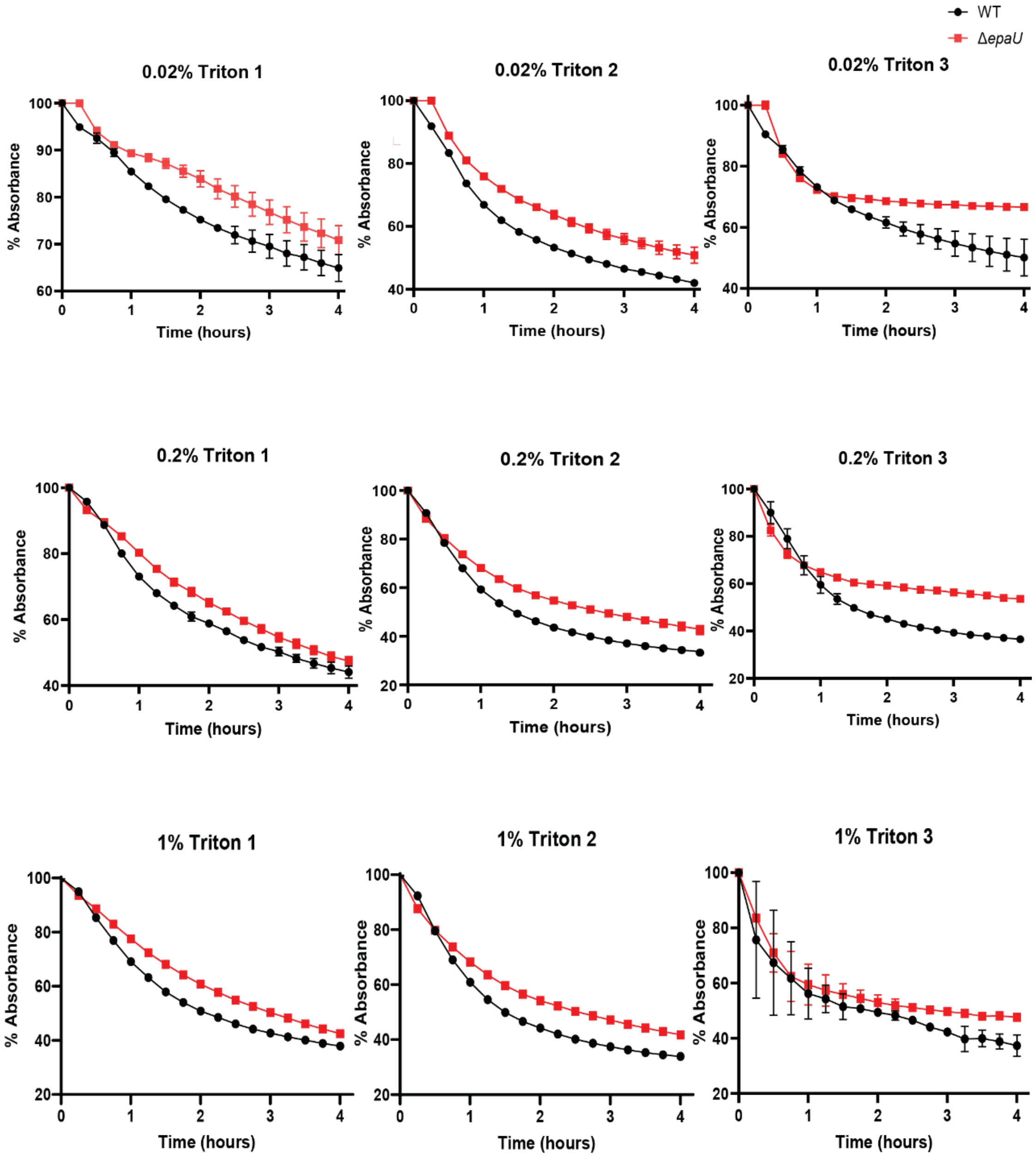

Figure S4. **Autolysis of WT (black line) vs Δ*epaU* (red line).**
Each biological replicate is shown at all tested Triton X-100 concentrations. Autolysis was monitored as the decrease in OD_{600} and normalized to the OD_{600} at time zero (100%). The error bars represent standard errors between technical repeats.

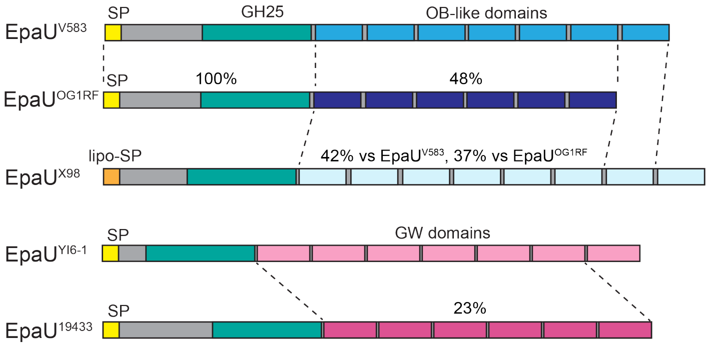

Figure S5. **The domain diagrams of EpaU autolysins from different *E. faecalis* strains.**

While all EpaU autolysins possess endo-N-acetylmuramidases catalytic domains (GH25 family), their cell wall-binding domains differ. The cell wall-binding domains of EpaU from V583, OG1RF and X98 strains have a variable number of repeat OB-like (Oligonucleotide/Oligosaccharide-Binding) domains (DUF5776) with limited sequence identity indicated by percentages. The cell wall-binding domains of EpaU from YI6-1 and ATCC 19433 strains have a variable number of repeat GW (Gly-Trp dipeptide) domains (PFAM 13457) with low sequence identity to each other and no homology to the structurally unrelated OB-like domains of EpaU from V583, OG1RF and X98 strains.

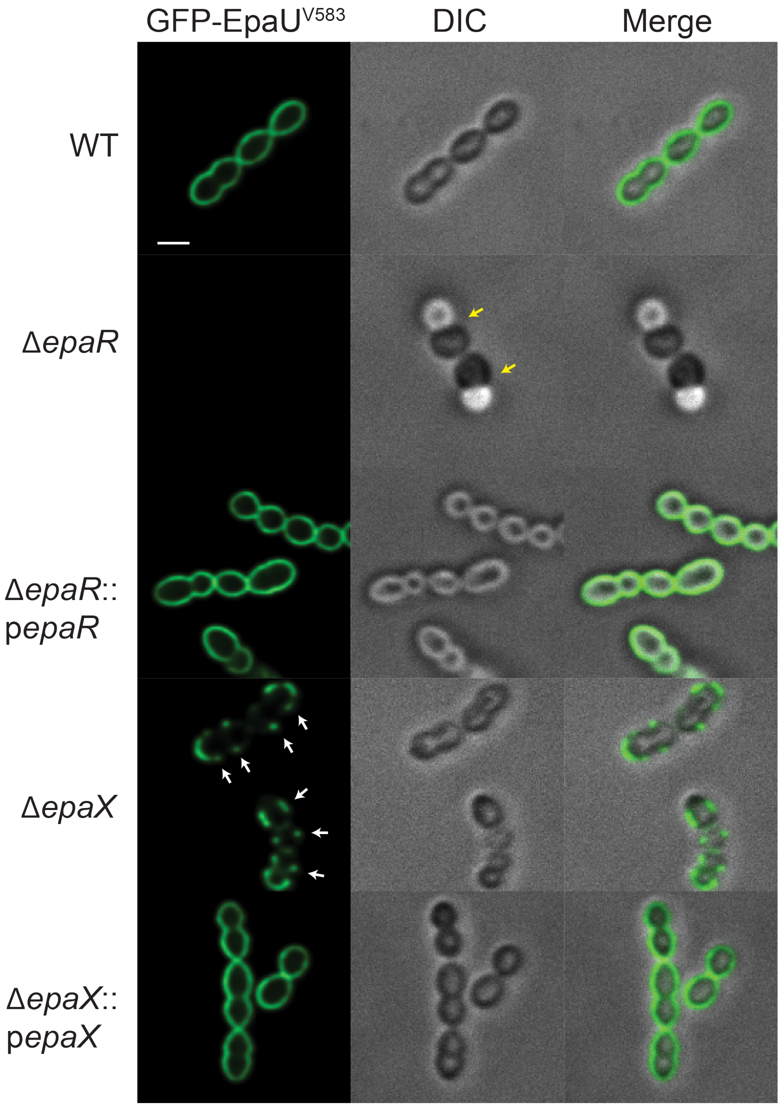

Figure S6. **Binding of 15.6 µg mL^{-1} GFP-EpaU^{V583} to *E. faecalis* cells.**
Exponential phase cells of the WT, Δ*epaR*, Δ*epaR*::p*epaR,* Δ*epaX* and Δ*epaX*::p*epaX* strains were incubated with 15.6 µg mL^{-1} GFP-EpaU^{V583} and examined with differential interference contrast (DIC) and fluorescent microscopy. White arrows indicate equatorial sites labeled by GFP-EpaU^{V583}. Yellow arrows indicate cell division defects. The experiments were performed independently three times and yielded the same results. A representative image from one experiment is shown. Scale bar is 1 µm.

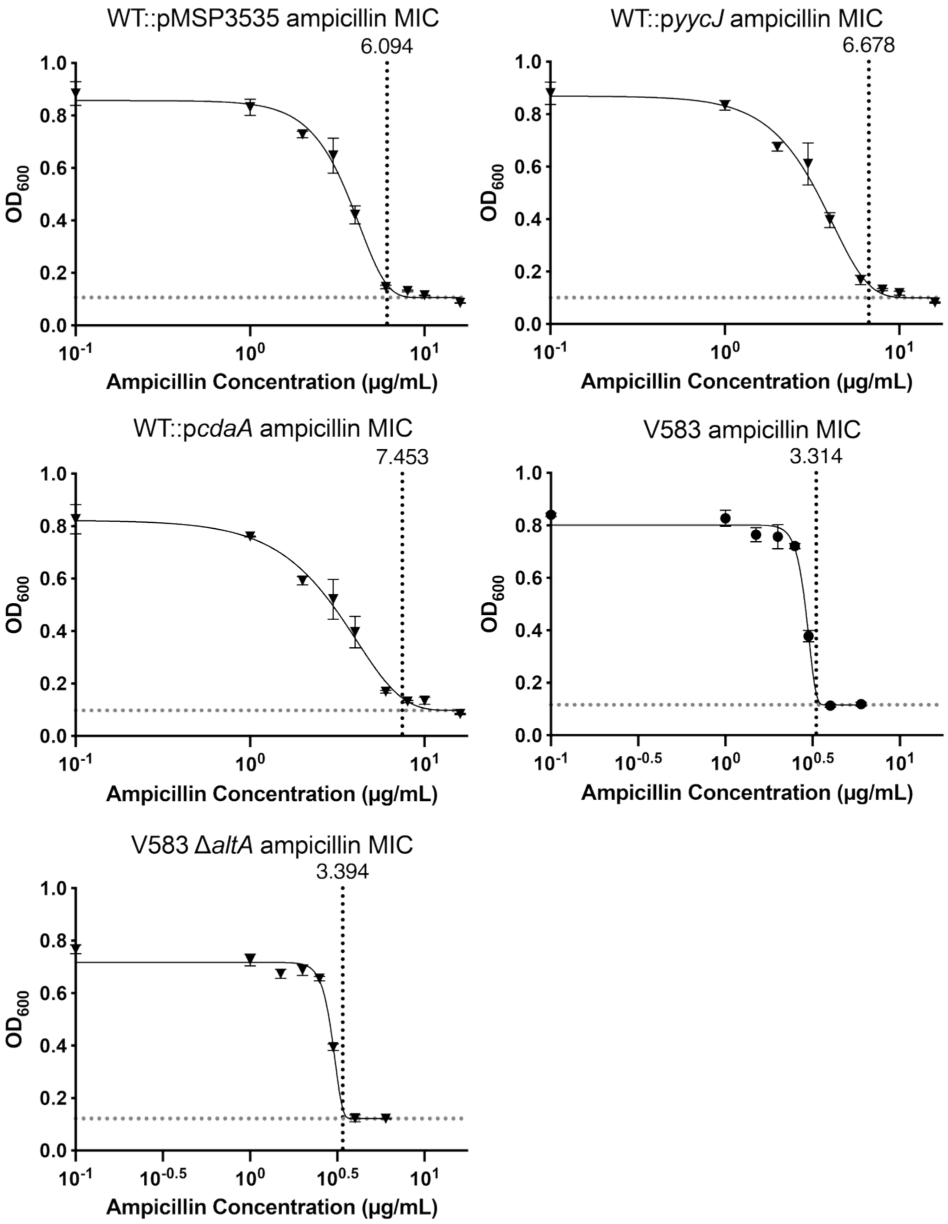

Figure S7. Best-fit curves for ampicillin resistance controls of wild-type expressing the empty pMSP3535 vector, and wild-type over-expressing both *yycJ* and *cdaA*. The WT::p*yycJ* was used as an additional control. The experiments with WT::pMSP3535, WT::p*yycJ*, and WT::p*cdaA* strains were performed with 25 ng/ml nisin added as an inducer. The Δ*altA* mutant was also measured alongside its V583 parent strain for reference. The vertical dotted line represents the best-fit MIC. MIC values are reported in μg/mL and were computed using Gompertz equation for MIC determination (2). Fits are the result of three independent biological repeats.

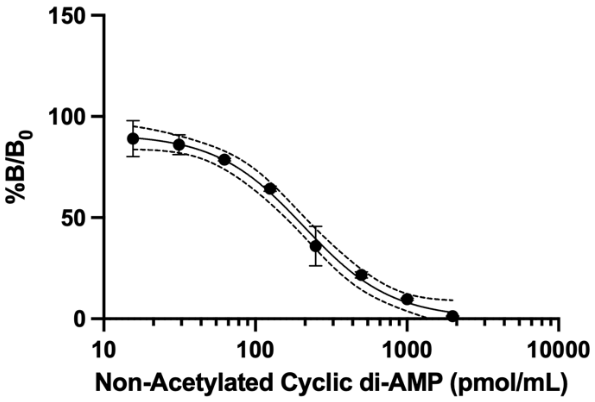

Figure S8. **Example standard curve for non-acetylated cAMP**.
Interpolation of unknowns was performed using an experimental standard curve measuring bound/maximum bound (%B/B_0) to known cAMP concentration for each assay run. 95% confidence intervals are shown as dashed lines using a sigmoidal variable slow semi-log fit with an R^2 = 0.9855. Each standard was measured in duplicate on each ELISA.

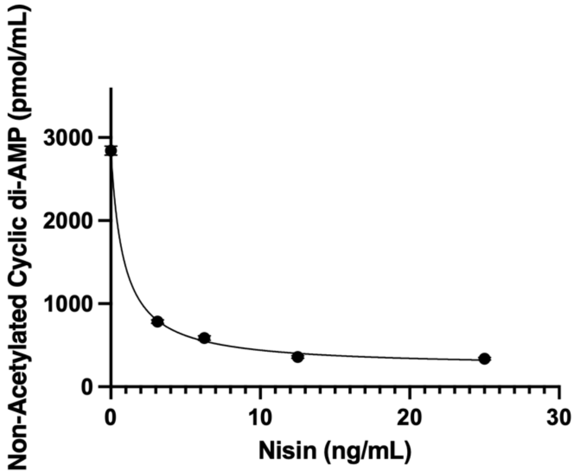

Figure S9. **EpaU induction with nisin shows a phenotype even at low protein levels**.
To correlate how much EpaU might be necessary to see an effect on c-di-AMP levels, various concentrations of nisin were used to induce EpaU in the Δ*epaU*::p*epaU* cells. Intracellular c-di-AMP concentration was measured using an ELISA. Levels of nisin far below the concentration for EpaU induction suppress c-di-AMP synthesis, suggesting that even a small amount of EpaU can reverse the phenotype. All measurements were done with three biologically independent repeats, with the line representing the median value.

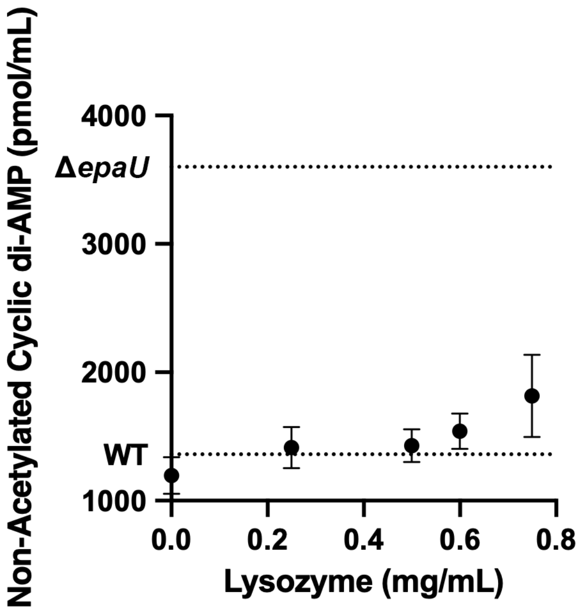

Figure S10. **Lysozyme cell wall challenge does not promote c-di-AMP level change.** Lysozyme was titrated into cultures during *E. faecalis* growth as outlined in Methods. Dashed lines mark the measured levels of WT and Δ*epaU* from Fig. 8C, respectively. Data are mean values ± S.D., n = 3 biologically independent experiments.

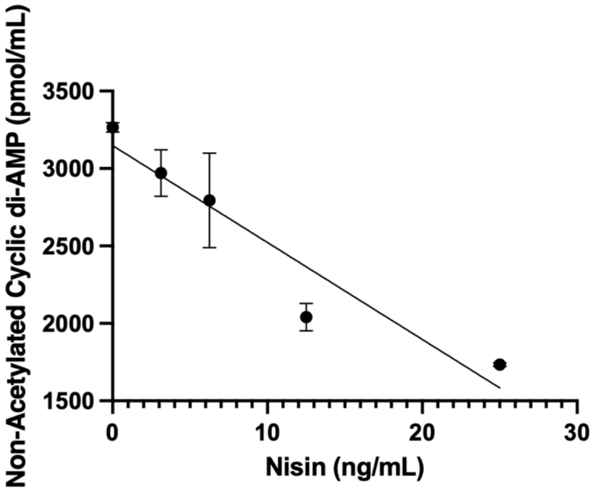

Figure S11. **Effect of expression of EpaU lacking the cell wall binding domain on c-di-AMP levels.**
A construct lacking the CWB domain, Δ*epaU*::p*epaU*ΔCWB, was induced with various levels of nisin and then assayed for intracellular c-di-AMP concentration using an ELISA. While high concentrations of nisin did show a phenotype more consistent with WT, lower levels of protein expression tend to look more like Δ*epaU*, suggesting that the CWB domain is critical for the function of EpaU at typical cellular concentrations, and activity of the catalytic only domain is only observed in the case of gross over-expression. All measurements are from three biologically independent experiments, with the trend-line representing a non-rigorous fit.

Table S1. MICs of other antibiotics tested between WT and Δ*epaU* strains.

**MIC**	**1316 (μg/mL)**	**95% CI (μg/mL)**	**Δ*epaU* (μg/mL)**	**95% CI (μg/mL)**
**Ampicillin**	6.986	6.390 to 7.714	10.61	9.322 to 12.38
**Penicillin**	1.833	1.616 to 2.104	1.940	1.672 to 2.288
**Fosfomycin**	11.39	10.65 to 12.34	12.66	ND[a] to 17.35
**Vancomycin**	2.828	2.638 to 3.050	3.062	2.696 to 3.547
**Tunicamycin**	1.110	1.056 to 1.170	1.008	ND
**Daptomycin**	9.731	8.514 to 11.29	10.37	8.689 to 12.54
**Ceftriaxone**	13.43	9.783 to 21.20	11.82	8.360 to 20.73

[a]Values marked not defined (ND) were unable to be computed by Prism and were reported by the program as interrupted calculations.

Table S2. Best-fit values for ampicillin resistance of other control strains.

**Best-fit values**	**WT::pMSP3535**	**WT::p*yycJ***	**WT::p*cdaA***	**V583**	**Δ*altA***
logMIC	0.7849	0.8246	0.8723	0.5204	0.5308
Slope	6.325	4.836	3.820	24.19	23.28
Bottom	0.1059	0.1005	0.09737	0.1158	0.1217
Span	0.7509	0.7687	0.7250	0.6858	0.5956
MIC	6.094	6.678	7.453	3.314	3.394

**95% CI**	**WT::pMSP3535**	**WT::p*yycJ***	**WT::p*cdaA***	**V583**	**Δ*altA***
logMIC	0.7381 - 0.8365	0.7670 - 0.8927	0.7964 - 0.9670	0.5052 - 0.5418	0.5098-0.5612
Slope	5.068 - 8.091	3.875 - 6.150	3.012 - 4.939	17.54 - 35.48	15.87-37.52
Bottom	0.07909 - 0.1314	0.06663 - 0.1306	0.05418 - 0.1331	0.08658-0.1451	0.09566-0.1478
Span	0.7058 - 0.7993	0.7141 - 0.8289	0.6603 - 0.7982	0.6489 - 0.7231	0.5627-0.6290
MIC	5.471 - 6.863	5.847 - 7.811	6.258 - 9.268	3.200 - 3.482	3.234 - 3.641

Best-fit values for ampicillin resistance of WT::pMSP3535, WT::p*yycJ*, Δ*altA*, and WT::p*cdaA* and V583 control strains. MIC reported in μg/mL; slope, bottom, and span are parameters from Gompertz equation for MIC determination (2). Fits are the result of three independent biological repeats.

Table S3. Strains and plasmids used in this study.

Strain or plasmid	Description [a]	Reference
Enterococcus faecalis		
VE14089	*E. faecalis* V583-derived strain cured of plasmids	(3)
V583	Vancomycin-resistant clinical isolate	(4)
Δ*epaR*	*epaR* deletion mutant in VE14089 strain	This study
Δ*epaX*	*epaX* deletion mutant in VE14089 strain	This study
Δ*epaR*::p*epaR*	Δ*epaR* complemented with p*epaR* carrying *E. faecalis epaR*, Cam^R	This study
Δ*epaX*::p*epaX*	Δ*epaX* complemented with p*epaX* carrying *E. faecalis epaX*, Cam^R	This study
OG1RF	spontaneous mutant of OG1 strain, resistant to rifampicin and fusidic acid	(5)
YI6-1	Clinical isolate YI6 cured of plasmid pYI6	(6)
ATCC 19433	NCTC 775	(7)
X98	fecal isolate (MLST 19)	(8)
Δ*cpsC*	*cpsC* deletion mutant in V583 strain	(9)
Δ*atlA*	*atlA* deletion mutant in V583 strain	(10)
Δ*epaU*	*epaU* deletion mutant in VE14089 strain	This study
Δ*epaU*::p*epaU*	Δ*epaU* complemented with p*epaU* carrying *E. faecalis epaU*, Er^R	This study
Δ*epaU*::pMSP3535	Δ*epaU* with empty complementation plasmid pMSP3535, Er^R	This study
VE14089::p*cdaA*	WT with p*cdaA* carrying *E. faecalis cdaA*, Er^R	This study
VE14089::p*yycJ*	WT with p*yycJ* carrying *E. faecalis yycJ*, Er^R	This study
Streptococcus mutans		
Δ*rgpG*	*rgpG* deletion mutant (has a nonpolar spectinomycin resistance cassette inserted in *rgpG*), $Spec^R$	(11)
Escherichia coli		
NovaBlue	*E. coli* strain for cloning	Novagen
MC1061	*E. coli* strain for cloning	(12)
LOBSTR(DE3)pRARE2	*E. coli* strain for protein expression; Cam^R	Kerafast
Plasmids		
pDC123	*E. coli*-*streptococcus* shuttle vector, JS-3 replicon, chloramphenicol resistance cassette, Cam^R	(13)
pLT06	Vector for making mutant *E. faecalis* strains, Cam^R	(9)
pMSP3535	Vector for nisin-controlled protein expression in *E. faecalis*, Er^R	(14)
pRSF-NT	Vector for protein expression in *E. coli* with N-terminal His_6 tag followed by a TEV cleavage site, Kan^R.	(15)
pKV1620	pLT06-based vector for *epaR* deletion, Cam^R	This study
pKV1694	pLT06-based vector for *epaX* deletion, Cam^R	This study

pKV1667	pLT06-based vector for *epaU* deletion, Cam^R	This study
pKV1633	p*epaR*, pDC123-based vector for Δ*epaR* complementation, Cam^R	This study
pKV1634	p*epaX*, pDC123-based vector for Δ*epaR* complementation, Cam^R	This study
p*epaU*	pMSP3535-based vector for Δ*epaU* complementation, Er^R	This study
p*epaU* E262Q	Vector with the catalytic site mutant EpaU E262Q, Er^R	This study
p*epaU* ΔCWB	Vector with EpaU lacking the CWB domain, Er^R	This study
p*cdaA*	pMSP3535-based vector for nisin-inducible expression of diadenylate cyclase CdaA, Er^R	This study
p*yycJ*	pMSP3535-based vector for nisin-inducible expression of a putative metallo hydrolase YycJ (EF1197), Er^R	This study
pKV1606	pRSF-NT-based vector for expression of sfGFP-$EpaU^{V583}$ C-terminal domain fusion, Kan^R	This study
pKV1608	pRSF-NT-based vector for expression of $EpaU^{V583}$ C-terminal domain, Kan^R	This study
pKV1695	pRSF-NT-based vector for expression of sfGFP-$EpaU^{OG1RF}$ C-terminal domain fusion, Kan^R	This study
pKV1696	pRSF-NT-based vector for expression of full-length $EpaU^{V583}$ without signal peptide and N-terminal IDR, Kan^R	This study

Table S4. Primers used in this study.

Primer	Sequence[a]	Genetic manipulations
epaR_F_BamH	CGCGGATCCGCGCGATTGGTGCAACGTTGATGT	construction of ΔepaR
epaR_R1_Not	ACTAGCGCGGCCGCTTGCTCCCCGTCTTGCATTACTCCATTCAC	
epaR_F1_Not	GGAGCAAGCGGCCGCGCTAGTGGATTGGATGAGTCTGAAGAACG	
epaR_R_EcoR	CCGGAATTCCGGTCCACTTTTCCTTTTCCTCCCT	
epaR_check_F	GAAACCGCTGGGGACACATA	verification of ΔepaR
epaR_check_R	TCTGGTTGAATTTCAAAATTTGAGACT	
2170_BamH_F	GTAGGATCCATGCGAATATTTCGATCTGGG	construction of ΔepaX
2170_armR	TAAAATCATTGTTTTTCTAAATATTTTTCTACATTGTATACAGGAAC	
2170_armF	AGAAAAATATTTAGAAAAACAATGATTTTACTTATGTTTGGTCTTCC	
2170_EcoR_R	GTTGAATTCCGAAAACAATAGCACCACAG	
2170_chF	GAGAAGCCACCGTCCAAG	verification of ΔepaX
2170_chR	CACCTGACAATAGCATAATTATCAAGG	
2174_BamH_F	GAGAGGATCCAGATTATGAAAAATTGAAAGTGC	construction of ΔepaU
2174_R2	TATTAGCACTAAAACTATTTAATAATACAGTACAAATTAACATACC	
2174_F2	ATTATTAAATAGTTTTAGTGCTAATAAGTGGTATGTAGCAAAAGTG	
2174_EcoR_R	GAATGAATTCGAATGAGCTGCTACAGGATATTAAC	
2174_checkF	TGAAATAAAGGTATGAGATAATAATTGGTG	verification of ΔepaU
2174_checkR	AAAAACACCTTTGTCGAACTCG	
2177_Xba	CTATCTAGAATTACTGGAAGTGAAGAGAGGAAG	construction of p*epaR*
2177_BamH	CTTGGATCCTTAACGGTAAATTCTAATAGCATTCTCTTTAC	
2170_Xba	CTATCTAGAATTTTAGAAGGTAGCGGAAATTGATATG	construction of p*epaX*
2170_BamH	CTTGGATCCTTAATCTATTGTTTTTTCTATATATTTTCTTTTCAATTTAATTGG	
epaU-BamH-F	CCAGGATCCATATTATTAAATATATAAGGAGAGTTTCATG	construction of p*epaU*
epaU_Xho	CTTCTCGAGCTTAACCCACTTTTGCTAC	
epaU_E262Q_F	TAATGATGCGCAAGATCCAACGCTTACAAAC	construction of p*epaU* E262Q
epaU_E262Q_R	GTTGGATCTTGCGCATCATTAAACATTATGG	
epaU_FLAG_F	CGGTGATTATAAAGATCATGATATCGATTACAAGGATGACGATGACAAGTAAGGGCCTGACTTAAGTAAG	construction of p*epaU* ΔCBD
epaU_FLAG_R	ATATCATGATCTTTATAATCACCGTCATGGTCTTTGTAGTCACTACTATTACCCATATTTAAAAAATTAC	
cdaA_BamH	GATGGATCCGGTATACTTGTTAGGAAATCTTGAG	construction of p*cdaA*
cdaA_Xho	ATCCTCGAGTCATTTGCGTTTAACCCCC	
mbl_Bgl2	GATTAGATCTGAAACACGTTAATGTTGTAATGTAG	construction of p*yycJ*
mbl_Xho	ATCCTCGAGCTAAATTTGAAATAACTCTGAGGC	
sfGFP_Nco	GAGACCATGGCTAGCAAAGGAGAAGAACTTTTC	

gfp_2174_R	CTTACTTAAGTCAGGCCCACTACCTCCACCTTTGTAGAGC	construction of pKV1606
gfp_2174_F	GCTCTACAAAGGTGGAGGTAGTGGGCCTGACTTAAGTAAG	
2174_Hind	CTCAAGCTTAACCCACTTTTGCTACATAC	
2174_374Nco	GAGACCATGGGGCCTGACTTAAGTAAGTATTAC	construction of pKV1608
2174_Hind	CTCAAGCTTAACCCACTTTTGCTACATAC	
sfGFP_Nco	GAGACCATGGCTAGCAAAGGAGAAGAACTTTTC	construction of pKV1695
U_og1rf-R1	GGAATTGCCACCTCCACCTTTGTAGAGC	
U_og1rf-F1	GCTCTACAAAGGTGGAGGTG	
U_og1rf-R	GGTGCTCGAGTGCGGCCGCAAGCTTAATTC	
2174_113BspH	GAGATCATGACCATTGACCAAGATGAAGC	construction of pKV1696
2174_Hind	CTCAAGCTTAACCCACTTTTGCTACATAC	

[a] Restriction sites are underlined.

References

1. Nambiar A, Forsyth JM, Liu S, Maslov S. 2024. DR-BERT: A protein language model to annotate disordered regions. Structure 32:1260-1268.
2. Lambert RJ, Pearson J. 2000. Susceptibility testing: accurate and reproducible minimum inhibitory concentration (MIC) and non-inhibitory concentration (NIC) values. J Appl Microbiol 88:784-790.
3. Rigottier-Gois L, Alberti A, Houel A, Taly JF, Palcy P, Manson J, Pinto D, Matos RC, Carrilero L, Montero N, Tariq M, Karsens H, Repp C, Kropec A, Budin-Verneuil A, Benachour A, Sauvageot N, Bizzini A, Gilmore MS, Bessieres P, Kok J, Huebner J, Lopes F, Gonzalez-Zorn B, Hartke A, Serror P. 2011. Large-scale screening of a targeted Enterococcus faecalis mutant library identifies envelope fitness factors. PLoS One 6:e29023.
4. Sahm DF, Kissinger J, Gilmore MS, Murray PR, Mulder R, Solliday J, Clarke B. 1989. In vitro susceptibility studies of vancomycin-resistant Enterococcus faecalis. Antimicrob Agents Chemother 33:1588-1591.
5. Dunny GM, Brown BL, Clewell DB. 1978. Induced cell aggregation and mating in Streptococcus faecalis: evidence for a bacterial sex pheromone. Proc Natl Acad Sci U S A 75:3479-3483.
6. Ike Y, Clewell DB. 1992. Evidence that the hemolysin/bacteriocin phenotype of Enterococcus faecalis subsp. zymogenes can be determined by plasmids in different incompatibility groups as well as by the chromosome. J Bacteriol 174:8172-8177.
7. Jones D, Shattock PM. 1960. The location of the group antigen of group D Streptococcus. J Gen Microbiol 23:335-343.
8. Palmer KL, Carniol K, Manson JM, Heiman D, Shea T, Young S, Zeng Q, Gevers D, Feldgarden M, Birren B, Gilmore MS. 2010. High-quality draft genome sequences of 28 Enterococcus sp. isolates. J Bacteriol 192:2469-2470.
9. Thurlow LR, Thomas VC, Hancock LE. 2009. Capsular polysaccharide production in Enterococcus faecalis and contribution of CpsF to capsule serospecificity. J Bacteriol 191:6203-6210.
10. Thomas VC, Hiromasa Y, Harms N, Thurlow L, Tomich J, Hancock LE. 2009. A fratricidal mechanism is responsible for eDNA release and contributes to biofilm development of Enterococcus faecalis. Mol Microbiol 72:1022-1036.
11. Zamakhaeva S, Chaton CT, Rush JS, Ajay Castro S, Kenner CW, Yarawsky AE, Herr AB, van Sorge NM, Dorfmueller HC, Frolenkov GI, Korotkov KV, Korotkova N. 2021. Modification of cell wall polysaccharide guides cell division in Streptococcus mutans. Nat Chem Biol 17:878-887.
12. Casadaban MJ, Cohen SN. 1980. Analysis of gene control signals by DNA fusion and cloning in Escherichia coli. J Mol Biol 138:179-207.
13. Chaffin DO, Rubens CE. 1998. Blue/white screening of recombinant plasmids in Gram-positive bacteria by interruption of alkaline phosphatase gene (phoZ) expression. Gene 219:91-99.
14. Bryan EM, Bae T, Kleerebezem M, Dunny GM. 2000. Improved vectors for nisin-controlled expression in Gram-positive bacteria. Plasmid 44:183-190.
15. Korotkov KV, Delarosa JR, Hol WGJ. 2013. A dodecameric ring-like structure of the N0 domain of the type II secretin from enterotoxigenic Escherichia coli. J Struct Biol 183:354-362.
